# Genetic Correlation Between Fe and Zn Biofortification and Yield Components in a Common Bean (*Phaseolus vulgaris* L.)

**DOI:** 10.3389/fpls.2021.739033

**Published:** 2022-01-03

**Authors:** Santiago Diaz, Jose Polania, Daniel Ariza-Suarez, Cesar Cajiao, Miguel Grajales, Bodo Raatz, Stephen E. Beebe

**Affiliations:** Bean Program, Crops for Health and Nutrition Area, Alliance Bioversity International – CIAT, Cali, Colombia

**Keywords:** QTL, iron, zinc, bean, biofortification

## Abstract

Common bean (*Phaseolus vulgaris* L.) is the most important legume for direct human consumption worldwide. It is a rich and relatively inexpensive source of proteins and micronutrients, especially iron and zinc. Bean is a target for biofortification to develop new cultivars with high Fe/Zn levels that help to ameliorate malnutrition mainly in developing countries. A strong negative phenotypic correlation between Fe/Zn concentration and yield is usually reported, posing a significant challenge for breeders. The objective of this study was to investigate the genetic relationship between Fe/Zn. We used Quantitative Trait Loci (QTLs) mapping and Genome-Wide Association Studies (GWAS) analysis in three bi-parental populations that included biofortified parents, identifying genomic regions associated with yield and micromineral accumulation. Significant negative correlations were observed between agronomic traits (pod harvest index, PHI; pod number, PdN; seed number, SdN; 100 seed weight, 100SdW; and seed per pod, Sd/Pd) and micronutrient concentration traits (SdFe and SdZn), especially between pod harvest index (PHI) and SdFe and SdZn. PHI presented a higher correlation with SdN than PdN. Seventy-nine QTLs were identified for the three populations: 14 for SdFe, 12 for SdZn, 13 for PHI, 11 for SdN, 14 for PdN, 6 for 100SdW, and 9 for Sd/Pd. Twenty-three hotspot regions were identified in which several QTLs were co-located, of which 13 hotpots displayed QTL of opposite effect for yield components and Fe/Zn accumulation. In contrast, eight QTLs for SdFe and six QTLs for SdZn were observed that segregated independently of QTL of yield components. The selection of these QTLs will enable enhanced levels of Fe/Zn and will not affect the yield performance of new cultivars focused on biofortification.

## Introduction

Common bean (*Phaseolus vulgaris* L.) is one of the most important legumes for human consumption and is cultivated worldwide, particularly in tropical and subtropical countries of Africa and America (Broughton et al., 2003). Cultivated common bean is organized in two major gene pools, Mesoamerican and Andean, which resulted from independent domestication events, generating landraces with diverse seed quality, nutritional traits, resistance to diseases, tolerance to abiotic stress, morphologic, and agronomic traits (Schmutz et al., 2014). Bean has been documented as a highly valuable food for the human diet, an accessible and relatively inexpensive source of proteins and micronutrients, such as iron and zinc (Beebe, 2012).

Micronutrient deficiencies are among the most common global nutritional problems, affecting half of the population of the world, especially women and children in developing countries (Bouis and Welch, 2010). Fe and Zn deficiencies are among the main common causes of micromineral malnutrition worldwide. In humans, Fe is essential for several cellular mechanisms (Steinbicker and Muckenthaler, 2013). Iron Deficiency Anemia (IDA), a pathology associated with scarce iron intake and absorption, affects a third of the population of the world and has been associated with several chronic diseases (Lopez et al., 2016). Zn has a key role in regulating growth and in the functioning of the immune system (Chasapis et al., 2012), and its deficiency can contribute to infections (Hambidge, 2000).

Wide phenotypic variability has been reported in bean in terms of Fe and Zn accumulation, with seed Fe concentration from 35 to 90 mg/kg and seed Zn concentration from 20 to 54 mg/kg (Beebe et al., 2000), these ranges are higher than those of other main staple crops, such as rice, wheat, and maize (Petry et al., 2015). For this reason, common bean is one of the crops targeted for biofortification. A cup of beans supplies 25% of Fe and 15% of Zn to the recommended daily allowance in the human diet, but breeding programs have the potential to develop genotypes with two to three times more Fe and Zn concentrations (Cichy et al., 2009).

Bouis (2003) reported that biofortification breeding has multiple positive effects, such as more efficiency in nutrients, fertilizer, and irrigation demands, and can increase disease resistance and yield. However, some difficulties have been described in bean breeding for micronutrient concentration, mainly a strong negative correlation between Fe and Zn concentration and yield. Source genotypes with high iron contents (>100 mg/kg) have been identified, but most of these lines have poor agronomic performance (Raatz, 2018). For this reason, a major breeding objective is overcoming the negative correlation between the micronutrient concentrations with yield components and finding the optimum balance between these traits.

While the phenotypic variability for Fe and Zn in common bean has been utilized in breeding programs with some success, the development of genomic tools will likely be helpful to reach the goals of biofortification (Beaver and Osorno, 2009). Currently, the genetic architecture of Fe and Zn accumulation in seed in common bean has been the object of several studies (Izquierdo et al., 2018), usually using Quantitative Trait Loci (QTL) analysis to find genomic regions related to the genetic control of these traits. Nevertheless, molecular markers have not been implemented in bean breeding programs focused on mineral concentration, as parental materials employed for populations are often distant from elite breeding materials, and the QTLs found with major effects are scarce and cannot be extrapolated to other populations (Raatz, 2018).

Another factor to consider in biofortification breeding is the accompanying agronomic traits, such as drought tolerance. Hummel et al. (2018) predicted a large part of current common bean growing areas to become unsuitable for cultivation by 2050, generating reductions in yield for current varieties and potentially affecting the nutritional quality, especially the iron levels of the crop, if current climate change trends continue. Drought is the main cause of yield loss worldwide, and its incidence and duration are expected to increase due to climate change (Lauer et al., 2012). Drought stress is a principal abiotic constraint that affects beans, causing losses close to 60% in production (Beebe et al., 2008).

The objective of this study was to investigate the correlation of genetic architecture of Fe and Zn accumulation and yield components in a common bean under irrigated and drought conditions using QTL and Genome-Wide Association Studies (GWAS) analysis in three biofortified bi-parental Mesoamerican populations and to identify genomic regions associated with yield components, micromineral accumulation, and their possible relationship.

## Materials and Methods

### Plant Material

In this study we used three Mesoamerican bi-parental populations of common bean (1) SCR16 × SMC40, (2) SMC44 × SCR9, and (3) SMC33 × SCR16. Parents SCR16 and SCR9 are improved Mesoamerican lines from the International Center of Agricultural Tropical (CIAT) with traits related to drought tolerance and resistance to bean common mosaic virus (*BCMV*) and bean golden yellow mosaic virus (*BGYMV*). The parents SMC40, SMC44, and SMC33 are improved Mesoamerican lines with a high accumulation of Fe and Zn in the seed. Each population consists of 100 recombinant inbred lines (RILs). Populations were advanced by single seed descent, and families from which individual plants were drawn were evaluated for iron and zinc concentration, and yield components in the F3, F4, F5, F6, F7, and F8 generations (Table 1).

**TABLE 1:** Description of trials used in the experiment.

Year	Condition	Season^1^	Trial name	Generation
2011	Drought	B	2011_Dro_B	F2.3
2012	Drought	B	2012_Dro_B	F2.4
2013	Irrigated	A	2013_Irr_A	F3.5
	Drought	B	2013_Dro_B	F3.5
2014	Irrigated	A	2014_Irr_A	F4.6
	Drought	B	2014_Dro_B	F5.7
	Irrigated	B	2014_Irr_B	F5.7
2015	Irrigated	A	2015_Irr_A	F6.8

*^1^Sowing seasons per year. A, first semester; B, second semester.*

### Field Trials Design and Phenotyping

The three bi-parental populations were planted at the CIAT experimental station in Palmira, Colombia (altitude of 1,000 m.a.s.l., latitude 3° 32′ N and longitude 76° 18′ W). The experimental design used for this study was an augmented design with three blocks in which five different check lines (SER118, SER16, Tio Canela 75, SEN56, and DOR390), and each parent for each population was evenly distributed across the field. The experimental units in these trials were row plots of 2.22 m^2^ for each line. The experiments were conducted in 5 years (2011, 2012, 2013, 2014, and 2015) in four irrigated trials and four drought trials, for a total of eight trials, with data taken on families as the RILs were advanced from F2.3 to F6.8 (Table 1). For more information about the climatic conditions see Supplementary Figure 1.

The seed was harvested manually by plot upon maturity (75–80 days after sowing). This was estimated when 50% of plants of the plot had at least one pod losing its green pigmentation. At the time of the harvest, 0.5 m per plot were collected separately to measure the number of pods per m^2^ (pod number, PdN), number of seeds per m^2^ (seed number, SdN), 100 seed weight (100SdW) was obtained estimating the weight of the seeds per plot and corrected to the moisture content in the seed of 14%, number of seeds per pod (Sd/Pd), and pod harvest index (PHI, %) defined as the ratio between seed weight and total pod weight, such as seed (Assefa et al., 2013). In 2011, only PHI was measured. Additional information on the description of phenotypic traits can be found in ‘‘Traits Dictionaries for Fieldbook Development.’’^1^.

### Fe and Zn Quantification

The samples to evaluate iron and zinc concentrations in the seed (SdFe and SdZn, mg/kg) were prepared according to the method described by Stangoulis and Sison (2008). In brief, we collected 5 g of seed and cleaned using a cloth dampened with high-purity water. Later, seeds were dried to 7–8% moisture content in an oven at 60°C. Finally, samples were ground with a grinding mill Retsch Mixer Mill MM 400 using grinding jars and zirconium balls (Retsch GmbH & Co KG, Germany) to avoid iron and zinc contamination. To quantify SdFe and SdZn a Energy Dispersive X-ray Fluorescence machine (EDXRF) Bruker S2 PUMA (Bruker Corporation, Billerica, MA, United States) was used. The quantification was developed according to the method described by Guild et al. (2017a). In summary, the EDXRF analysis conditions were as follows. Atmosphere: air, X-ray tube: palladium (50 W), voltage: 40 kV, current: 240 μA, peak detected: Kα, acquisition time: 30 s, tube filter: Al 500 nm, and detector: silicon drift detector. Analyses were conducted in supplied sample cups prepared as reported previously by Guild et al. (2017a). Sample cups were cleaned and prepared before each analysis to minimize cross-contamination between samples. For the calibration, we used 10 custom-made 40 mm diameter glass disks (FLUXANA GmbH & Co., Germany) with a range of nominal Fe and Zn levels to establish a non-matrix-matched calibration. SdFe and SdZn were measured in all trials except in the 2011.

### Data Analysis

Statistical analysis of the phenotypic data was conducted with statistical software R (v3.3.2.). Best linear unbiased estimators and predictors (BLUEs/BLUPs) were calculated for each trait and each trial using the “lme4” Package (Bates et al., 2015). The data were modeled using the following equation:


$${\text{y}}_{\mathrm{mij}}=\mu +{\text{G}}_{m}+{\text{R}}_{i}+\varepsilon_{\mathrm{mij}}$$

where *y* is a vector with the phenotypic data, μ is the overall intercept, *G*_*m*_ is the effect of the *m*-th genotype, *R*_*i*_ is the effect of the *i*-th replication, and ε*_*mij*_* is the error term corresponding to *y*_*mij*_. In this model, *G*_*m*_ term effects were treated either as fixed (to calculated BLUEs) or random (to obtain an estimate of the genetic variance and calculated BLUPs). We assumed that every random term μ and the residual ε adjust to a normal distribution with mean 0 and independent variances μ∼ *N* (0, σ*^2^_*u*_I*) and ε∼ *N* (0, σ^2^_ε_ *I*).

To determine the proportion of the genetic variance controlling for evaluated traits, broad-sense heritability (H^2^) estimates were calculated using the method proposed by Cullis et al. (2006), using the equation below:


$${\text{H}}^{2}=1-\frac{\upsilon_{\mathrm{BLUP}}}{2\sigma_{\mathrm{genotype}}^{2}}$$

where υ_*BLUP*_ is the mean-variance of a difference of two BLUPs of genotypic effects, and $2\sigma_{genotype}^{2}$ is the genetic variance. The phenotypic correlation between traits measured was expressed as Pearson’s correlation coefficients among BLUEs for individual trials and cross-trials means, and their significance was tested using a two-tailed *t*-test.

### Genotyping

DNA extraction of the RILs and parents was developed on the F6.8 generation. For DNA extraction, seeds of each genotype were selected and germinated using a Calcium Sulfate 0.5-mM solution, the DNA was extracted from young leaves using liquid N^2^ and the urea buffer-based extraction miniprep protocol. The genotyping was performed using the *Ape*KI-based genotyping-by-sequencing (GBS) protocol (Elshire et al., 2011), and the GBS libraries were sequenced at the HudsonAlpha Institute for Biotechnology.^2^ Some lines were re-genotyped where seed quality was poor.

The mapping and variant calling processes for GBS reads are described in detail by Perea et al. (2016). In brief, the GBS reads were demultiplexed using the Next Generation Sequencing Eclipse Plugin (NGSEP; v3.3.2) (Tello et al., 2019). Adapters and low-quality bases from the raw sequencing data were trimmed using Trimmomatic (v0.36) (Bolger et al., 2014), and reads were aligned to the reference genome of *P. vulgaris* G19833 v2.1. (Schmutz et al., 2014) using Bowtie2 (v2.2.30) (Langmead and Salzberg, 2012) with default parameters. The variant calling process of RILs was performed using NGSEP following recommended parameters for GBS data (Perea et al., 2016), using the genotype calls of the parents (SMC44, SMC40, SMC33, SCR16, and SCR9) as reference. The resulting genotype matrix was filtered to remove genotype calls with quality below 40, remove markers with more than 6% heterozygote calls, minor allele frequency (MAF) below 0.02, and remove markers in repetitive regions of the reference genome (Lobaton et al., 2018). The process to construct a genotype matrix was carried out for each population separately and grouping the populations for GWAS analysis. The final matrices contained 1,650 markers in the SCR16 × SMC40 population, 3,033 markers in the SMC44 × SCR9 population, and 481 markers in the SMC33 × SCR16 population.

### Genetic Map Construction, Quantitative Trait Loci Analysis, and Genome-Wide Association Studies Analysis

For the construction of the genetic maps, the integrated tools of the IciMapping Software v4.1 (Meng et al., 2015) were used, employing the matrices above described. The genotypic data were grouped and anchored on their physical position. The default logarithm of odds (LOD) score (3.0) and rfs (0.35) were used as a maximum threshold value for grouping markers. The Kosambi genetic mapping function was chosen for genetic interval estimation using recombination frequency. The orders of markers per chromosome were set by their physical position, and distortion of segregated markers was exhibited according to segregation ratios. QTL analysis for the populations was conducted using the genetic maps of each population and the phenotypic BLUEs for each RIL. Detection of QTLs and estimation of genetic parameters for SdFe, SdZn, PHI, PdN, SdN, 100SdW, and Sd/Pd were performed using the inclusive composite interval mapping for additive effects (ICIM-ADD) procedure of the software IciMapping (v4.1) (Meng et al., 2015) with 10 cm windows and a sliding parameter of 1 cm. Significant QTL was considered by defining the LOD score at a genome-wide type I error rate of α = 0.05 after 1,000 permutation tests for each trait. The detected QTLs having high phenotypic variation explained (PVE) (>10%) were justified as major QTL (Amanullah et al., 2021). QTL hotspots were defined as significant loci genetically linked at <5 cm between QTL.

Genome-Wide Association Studies analysis was carried out using the R package GAPIT (v3.0) (Tang et al., 2016). The association analysis was conducted using a Compression Mixed Linear Model (CMLM) approach. This model accounts for population structure using the top four principal components as fixed effects. It also accounts for random polygenic effects with a kinship matrix as variance-covariance structure, calculated using the method proposed by VanRaden (2008) implemented in the GAPIT package. Significant associations were defined when the *p*-value was equal to or smaller than the Bonferroni-corrected threshold (1.79 × 10^–6^) (Johnson et al., 2010). Manhattan and quantile–quantile (Q-Q) plot graphics were made using the qqman R package (Turner, 2018).

## Results

### Phenotyping Variation for Traits Evaluated

**FIGURE 1:**
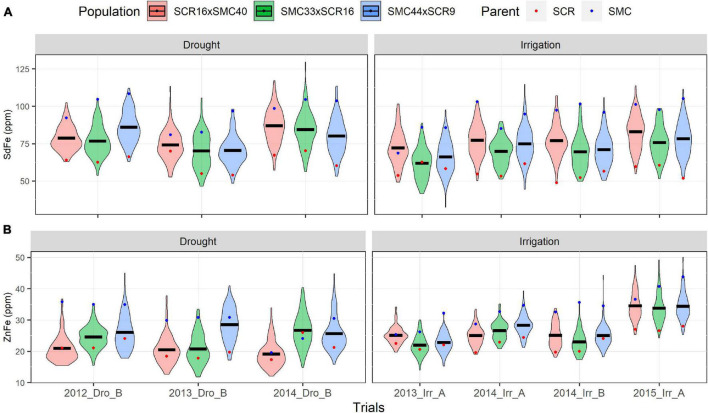
Phenotypic distribution of micronutrients content traits. Fe accumulation (SdFe) **(A)** and Zn accumulation (SdZn) **(B)** evaluated of 2012, 2013, 2014, and 2015 under drought and irrigated conditions for the SCR16 × SMC40, SMC33 × SCR16, and SMC44 × SCR9 populations.

**FIGURE 2:**
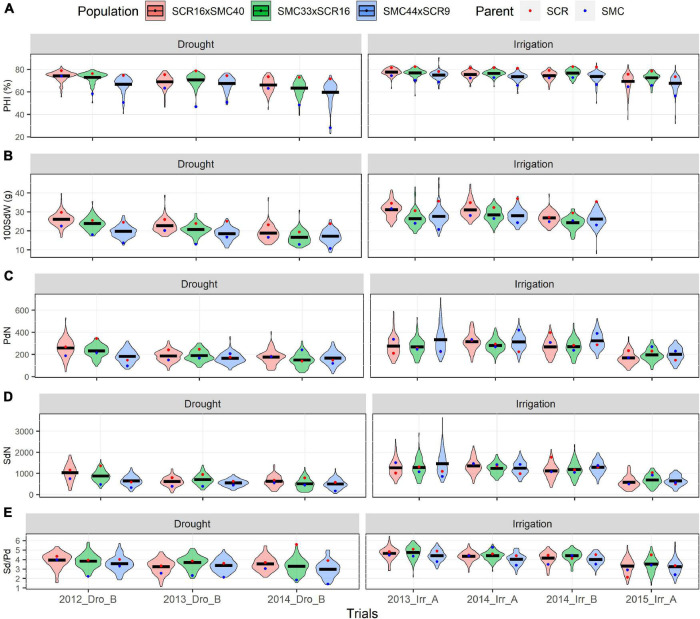
Phenotypic distribution of agronomics traits. Pod harvest index (PHI) **(A)**, 100 seed weight (100SdW) **(B)**, pod number (PdN) **(C)**, seed number (SdN) **(D)**, seed per pod (Sd/Pd), and **(E)** evaluated of 2012, 2013, 2014, and 2015 under drought and irrigated conditions for the SCR16 × SMC40, SMC33 × SCR16, and SMC44 × SCR9 populations.

Five elite lines of the CIAT bean-breeding program were used to develop three Mesoamerican bi-parental populations: (1) SCR16 × SMC40, (2) SMC33 × SCR16, and (3) SMC44 × SCR9. Families in several generations and F6.8 RILs were evaluated for a range of yield components and micronutrient concentrations of iron and zinc (SdFe and SdZn) in trials managed under irrigated and drought stress conditions. The phenotypic data show high and significant phenotypic variability among RIL in all traits evaluated (Supplementary Tables 1, 2 and Supplementary Figure 2). Transgressive segregation was also evident in the three populations with lower and higher values compared to parents in all traits. An effect of drought stress was not observed on SdFe, SdZn, PHI, and Sd/Pd in the populations evaluated. However, drought reduced PdN, SdN, and 100SdW by 29.5, 39.6, and 31.4%, respectively in all populations. The SdZn performance of SMC44 × SCR9 was better in most trials compared to the other populations (27.2 mg/kg on average), while SCR16 × SMC40 presented higher SdFe than the other two populations (78.3 mg/kg on average) (Figures 1, 2 and Supplementary Table 1).

The trial 2015_Irr_A presented the highest Zn accumulation for the three populations (>33 mg/kg for each population). In all trials, the SMC parents (SMC40, SMC44, and SMC33) presented a better performance in SdFe and SdZn than SCR parents (SCR9 and SCR16) (Figure 1). Conversely, SCR parents presented higher PHI, 100SdW, and Sd/Pd than the SMC parents (Figure 2). The broad-sense heritabilities for PHI, 100SdW, and Sd/Pd were high for the three populations: 0.70, 0.65, and 0.73 for SCR16 × SMC40; 0.79, 0.72, and 0.67 for SMC33 × SCR16; and 0.84, 0.79, and 0.72 for SMC44 × SCR9. In contrast, the heritabilities for the SdN and PdN were lower and not stable across trials (<0.53 for PdN and <0.47 for SdN). Heritabilities for micronutrient concentrations were intermediate ranging between 0.35 and 0.65 for SdFe and 0.58 and 0.70 for SdZn. SMC44 × SCR9 presented higher heritabilities compared to the other populations in most traits evaluated except for SdZn (Supplementary Table 1). Overall, the phenotypic datasets from field trials present a good quality for subsequent analyses.

**FIGURE 3:**
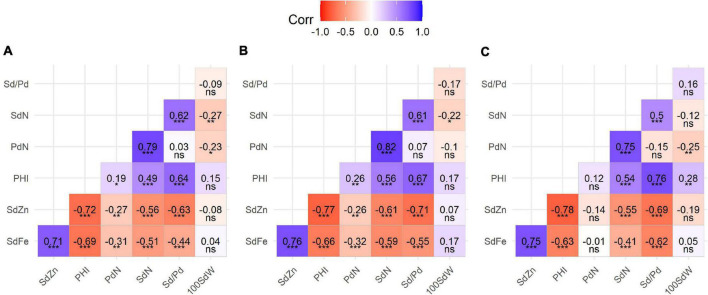
Pearson’s correlation coefficients between yield components and mineral concentration traits on three populations: **(A)** SCR16 × SMC40, **(B)** SMC33 × SCR16, and **(C)** SMC44 × SCR9. Significance of correlations indicated as ***p < 0.0001; **p < 0.001; *p < 0.01; ns, not significant. SdFe, seed iron; SdZn, seed zinc; PHI, pod harvest index; PdN, pod number; SdN, seed number; Sd/Pd, number of seeds per pod; 100SdW, 100 seed weight. Blue color indicates positive correlation and red color indicates negative correlation.

Pearson correlations between traits suggested two trait clusters according to the positive and generally significant correlations within clusters and negative correlations between them. On the one hand, the micronutrient concentrations of SdFe and SdZn form a group that is positively correlated among all populations (0.71–0.76). On the other hand, a second group is formed between PdN and SdN showing a strong positive correlation between them (0.75–0.82). However, PHI only shows a stable correlation with SdN and Sd/Pd (0.49–0.56 and 0.64–0.76, respectively). These agronomic traits (PHI, PdN, SdN, and Sd/Pd) show strong negative correlations with micronutrient concentrations, especially between PHI and SdFe (−0.63 to −0.69) and SdZn (−0.72 to −0.78). 100SdW only presented weak negative correlations with SdN and PdN (−0.25 and −0.27) (Figure 3 and Supplementary Table 3).

### Quantitative Trait Loci Analysis

Marker-trait associations were evaluated by QTL mapping using ICIM-ADD. In total, 79 QTLs for seven traits were identified for the three populations for eight individual trials: 14 for SdFe, 12 for SdZn, 13 for PHI, 11 for SdN, 14 for PdN, 6 for 100SdW, and 9 for Sd/Pd. Sixty-four QTLs of the 77 QTLs identified in this study are classified as “major QTL” (>10% PVE). Twenty-three hotspot regions were identified in which several QTLs were co-located (Table 2 and Supplementary Table 4). In GWAS analysis, results show low levels of significance (Supplementary Figures 3, 4).

**TABLE 2:** Five most important “major QTLs” identified by inclusive composite interval mapping for additive effects (ICIM-ADD), genetic and physical position, LOD, phenotypic variation explained and additivity, mapped for SdFe, seed iron; SdZn, seed zinc; PHI, pod harvest index; PdN, pod number; SdN, seed number; Sd/Pd, number of seeds per pod in SCR16 × SMC40, SMC33 × SCR16, and SMC44 × SCR9 populations evaluated in 2011, 2012, 2013, 2014, and 2015.

Trait	QTL name	Chr.	Pos. (cM)	Left marker	Right marker	LOD	PVE (%)	Add	Source
PdN	PdN2.1	2	6.0	Chr02_1904681	Chr02_1957517	3.2	13.4	27.9	SMC44
	PdN6.1	6	49.2	Chr06_28273144	Chr06_28493119	3.4	14.1	–23.1	SMC44
	PdN7.1	7	3.4	Chr07_444014	Chr07_528622	3.8	17.5	34.2	SMC33
	PdN7.2	7	14.5	Chr07_1475573	Chr07_1491651	8.5	13.2	–27.1	SMC44
	PdN8.2	8	53.0	Chr08_47670662	Chr08_51758938	3.4	14.7	28.5	SMC33
PHI	PHI2.1	2	34.4	Chr02_32651815	Chr02_33432574	5.8	17.2	–3.7	SMC33
	PHI3.1	3	7.1	Chr03_1303175	Chr03_2089482	2.9	13.2	1.8	SMC33
	PHI7.1	7	38.1	Chr07_8771144	Chr07_8994936	16.8	20.0	–11.4	SMC44
	PHI7.2	7	47.2	Chr07_21053823	Chr07_21106043	17.4	20.6	13.5	SMC44
	PHI7.3	7	80.8	Chr07_36965265	Chr07_36997111	3.7	14.4	–1.7	SCR16
Sd/Pd	Sd/Pd1.1	1	73.2	Chr01_50988725	Chr01_51029053	3.9	14.9	–0.3	SMC44
	Sd/Pd2.1	2	5.7	Chr02_1773128	Chr02_1863169	5.0	24.5	–0.4	SCR16
	Sd/Pd5.2	5	72.0	Chr05_39973668	Chr05_40035138	6.1	14.4	0.3	SCR16
	Sd/Pd7.2	7	38.1	Chr07_8771144	Chr07_8994936	8.3	14.9	–0.7	SMC44
	Sd/Pd10.1	10	35.5	Chr10_41705364	Chr10_41845732	5.6	16.7	–0.4	SMC44
SdFe	SdFe3.1	3	0.4	Chr03_128924	Chr03_178169	5.4	17.0	5.2	SMC44
	SdFe3.2	3	41.2	Chr03_32241300	Chr03_32532209	5.0	17.2	–6.3	SCR16
	SdFe6.3	6	42.6	Chr06_26259338	Chr06_26444453	10.4	19.5	5.0	SMC44
	SdFe6.4	6	52.6	Chr06_28871402	Chr06_28878273	3.0	15.4	5.7	SMC44
	SdFe7.1	7	81.5	Chr07_37050767	Chr07_37087581	3.6	14.4	4.6	SCR16
SdN	SdN2.1	2	5.7	Chr02_1773128	Chr02_1863169	3.3	15.7	–102.1	SCR16
	SdN7.1	7	3.4	Chr07_444014	Chr07_528622	3.6	14.9	177.5	SMC33
	SdN7.2	7	14.8	Chr07_1513753	Chr07_1548857	3.2	15.4	–81.2	SMC44
	SdN8.1	8	78.2	Chr08_60266587	Chr08_60277482	3.5	18.6	–109.2	SMC44
	SdN9.1	9	21.7	Chr09_20907648	Chr09_21865226	3.4	14.6	–84.5	SMC44
100SdW	100SdW6.1	6	30.0	Chr06_21622820	Chr06_21693622	3.8	13.6	–1.2	SCR16
	100SdW7.1	7	62.0	Chr07_30278406	Chr07_30493387	3.6	16.6	–1.6	SMC44
	100SdW7.2	7	74.6	Chr07_35056625	Chr07_35841130	2.9	10.1	1.6	SCR16
	100SdW8.1	8	90.0	Chr08_62043144	Chr08_62063701	4.6	11.5	–2.4	SMC44
	100SdW11.1	11	44.3	Chr11_5665676	Chr11_5713253	4.8	16.5	1.5	SCR16
SdZn	SdZn4.1	4	0.0	Chr04_22568	Chr04_55665	3.1	13.0	1.1	SMC44
	SdZn5.1	5	23.3	Chr05_3779357	Chr05_3956110	3.5	13.4	–1.4	SCR16
	SdZn5.1	5	34.3	Chr05_11706425	Chr05_11785008	5.6	18.7	2.7	SMC44
	SdZn8.2	8	92.1	Chr08_62309944	Chr08_62325409	3.5	13.0	3.6	SMC44
	SdZn10.1	10	11.7	Chr10_7899489	Chr10_8099574	7.2	18.2	–2.4	SCR16

*A complete list of significant QTL is available (Supplementary Table 3).*

*QTLs, Quantitative Trait Loci.*

Quantitative Trait Loci for Fe accumulation were identified on seven chromosomes (Pv03, Pv05, Pv06, Pv07, Pv08, Pv09, and Pv011). Ten major QTLs were identified; SdFe6.3 in the 2013_Dro_B trial was the QTL with the highest PVE (19.47%) with an additive positive effect of 5.04 mg/kg, while SdFe6.2 had an additive effect of 6.28 mg/kg. In all QTLs found for SdFe, the additive positive effects came from SMC parents (SMC44 and SMC33) except for SdFe7.1. SdFe3.1 was the only QTL expressed in more than one trial. QTLs for SdZn were located on Pv02, Pv03, Pv04, Pv05, Pv06, Pv07, Pv08, and Pv10, of which eight were major QTLs. SdZn5.1 was the QTL that explained the greatest phenotypic variance on the trait (18.73%), with an additive effect of 2.74 mg/kg. SdZn2.3 was the only QTL with an additive positive effect from an SCR parent (1.86 mg/kg), while the other QTL for SdZn with additive positive effect came from SMC44 or SMC33. SdZn8.2 presented a higher additive effect compared with other QTL (3.57 mg/kg) (Table 2 and Supplementary Table 4). SdZn10.1 was identified in two trials.

Twelve QTLs for PHI were found on Pv01, Pv02, Pv03, Pv05, Pv07, Pv08, Pv09, and Pv11. Seven of these QTLs were classified as major QTLs. PHI7.2 presented the highest PVE and highest positive additive effect (20.61% and 13.54%, respectively) in 2014_Dro_B under drought stress. All QTLs with positive additive effect for this trait originates in an SCR parent, except PHI7.3. On Pv07, three different loci controlling PHI were identified, and two of them were present in two trials. Eight major QTLs for SdN were identified with SdN8.1 as the QTL with the highest PVE (18.59%) and SdN7.1 with the highest additive effect (186.93 seeds). Eleven major QTLs for PdN were found. PdN7.1 was the QTL with the highest phenotypic variance of 17.55% and PdN4.1 had the highest additive effect of 65.33 pods. Most QTLs found for PdN with positive effect came from the SMC parent except for PdN6.1, Pdn7.2, and PdN8.1. Six QTLs for seed weight were identified on Pv06, Pv07, Pv08, and Pv11, 100SdW7.1 was the QTL with the highest PVE (16.62%) and 100SdW8.2 from SCR9 presented a high additive effect (2.44 g). The sources for all QTL with positive additive allelic effects for 100SdW were SCR parents except for 100SdW6.1. Seven major QTLs for seed per pod were identified. Sd/Pd2.1 presented the highest PVE (24.50%) and Sd/Pd7.2 showed the highest positive allelic effect (0.67 seeds per pod) (Table 2 and Supplementary Table 4).

Nine QTL hotspots for yield components (PHI, SdN, PdN, SdW, and Sd/Pd) were identified. Similarly, 14 QTL hotspots for yield components and micronutrient concentration were recognized, and only one QTL hotspot was observed controlling the concentration of both Fe and Zn independently from yield components (Supplementary Table 4). Fourteen QTLs related with SdFe were identified, of which seven QTLs are co-localized with QTL related with Sd/Pd, SdN, PdN, 100SdW, and PHI. Similarly, of 12 QTLs identified for SdZn, eight were co-localized with QTL related to the yield components (Table 2 and Supplementary Table 4). In most of these “hotpots” (Hotpots 4, 5, 6, 11, 12, 16, 18, 20, 21, 22, and 23), it was observed that the positive additive allelic effects for higher micronutrient concentrations were associated with the negative allelic effect for yield component traits. The high yield component QTL was usually donated by the SCR parent, while the high mineral QTL came from the SMC parent. These strong negative associations are especially visible between SdFe and SdZn with PHI and Sd/Pd, with two strong associations with 100SdW.

## Discussion

The populations analyzed showed ample phenotypic variability in terms of average accumulation of micronutrients: SCR16 × SMC40 (53.7–108.3 mg/kg for Fe and 17.5–36.8 mg/kg for Zn), SMC33 × SCR16 (50.0–101.6 mg/kg for Fe and 16.9–36.8 mg/kg for Zn) and SMC44 × SCR9 (49.2–107.5 mg/kg for Fe and 18.7–42.4 mg/kg for Zn) (Supplementary Table 1). These ranges of micronutrient concentrations are wider than those reported for other populations: Andean biparental populations AND696 × G19833 (39–79 mg/kg for Fe and 16–29 mg/kg for Zn) (Cichy et al., 2009); G21242 × G21078 (28–95 mg/kg for Fe and 17–49 mg/kg for Zn) (Blair et al., 2011); Mesoamerican biparental population G14519 × G4825 (35–97 mg/kg for Fe and 17–49 mg/kg for Zn) (Blair et al., 2010); intergene pool biparental populations DOR364 × G19833 (40–84 mg/kg for Fe and 17–42 mg/kg for Zn) (Blair et al., 2009); Cerinza × G10022 (54–100 mg/kg for Fe and 23–38 mg/kg for Zn) (Blair and Izquierdo, 2012); an African collection (23.6–78.3 mg/kg for Fe and 19–56.1 mg/kg for Zn) (Tryphone and Nchimbi-Msolla, 2010); an Andean collection (54.5–99.3 mg/kg for Fe and 21.6–39.7 mg/kg for Zn) (Katuuramu et al., 2018); a European collection (38.4–93.7 mg/kg for Fe and 18.9–43.6 mg/kg for Zn) (Caproni et al., 2020); and a multiparent advanced generation intercross (MAGIC) population (37.9–87.6 mg/kg for Fe and 18.5–39 mg/kg for Zn) (Diaz et al., 2020). Common bean has significant biofortification potential due to the wide phenotypic variability in terms of Fe and Zn concentration, producing improved lines that can accumulate >100 mg/kg of Fe and >45 mg/kg of Zn. This high accumulation capacity of micronutrients exceeds that of other legume species, such as chickpea (36–91 mg/kg of Fe), pea (26–91 mg/kg of Fe), and mung bean (35–87 mg/kg of Fe), and only surpassed by lentil (37–176 mg/kg of Fe) (Shiva et al., 2016; Upadhyaya et al., 2016; Ma et al., 2017; Jha and Warkentin, 2020; Kumar and Pandey, 2020; Sab et al., 2020).

Breeding programs need fast, accurate, and cheap methods for screening large numbers of breeding lines and germplasm for genotypes with high micronutrients contents. EDXRF is an alternative to inductively coupled plasma-optical emission spectrometry (ICP-OES), the most common tool used for measuring microelements (Guild et al., 2017b). The benefits of this method compared to ICP lie in minimal sample preparation before analysis, less labor time, and cost required [∼$0.15 per sample to X-ray diffraction (XRD) method]. In addition, it is a method with a high level of prediction. Guild et al. (2017a) reported values of prediction models of 96% for Fe and 93% for Zn compared with ICP reference concentration, with an SE of prediction of 2.5 mg/kg of Fe and 1.5 mg/kg of Zn with a maximum coefficient of variation of 5% for Fe and 3.5% for Zn.

The genetic basis underlying the micronutrient concentration have been widely studied in common bean using QTL analysis, and GWAS (Gonzalez et al., 2017; Izquierdo et al., 2018; Gunjača et al., 2021). QTL mapping is an excellent strategy for gene mapping in this case because only a bi-parental population and a small set of markers are necessary a bi-parental population and a small set of markers, which are utilized to identify the genomic regions that segregate with a trait. However, this strategy is usually low in resolution since only two alleles per locus are analyzed, and genetic recombination is limited (Islam et al., 2016). Traditional QTL mapping is highly dependent on the genetic diversity of the two parents, and the effects of the detected QTLs can vary between populations. For this reason, the validation is necessary in other populations. GWAS analysis did not show any significant association. This lacks correlation to the type of population used for the biofortification analysis, as biparental populations are more suited for QTL mapping (Diaz et al., 2021). The limitations of QTL analysis can be overcome using GWAS, which can narrow down the candidate regions using natural populations. However, its use in biparental populations presents problems by low events recombination typical of this type of populations and low allelic richness required for GWAS. On the other hand, GWAS is susceptible to report false-positive associations, and its results require validation. The number of markers used in the GWAS highly affects its results (Han et al., 2018). In this study, 14 QTLs for SdFe were identified, of which 9 QTLs have been previously reported. Astudillo et al. (2013) reported two QTLs co-located respectively to SdFe3.2 and SdFe11.2 in an inter-gene pool population; the positive allele for high iron in that study came from the Andean parental (G19833). In this study, SdFe6.3 showed the highest percentage of variance explained and was the most significant QTL identified for Fe. This QTL was identified previously in an Andean biparental population (Cichy et al., 2009), in an intergene pool population (Astudillo et al., 2013), in an Andean collection (Katuuramu et al., 2018), and a Meta-QTL analysis using different populations (Izquierdo et al., 2018). Other QTLs, such as SdFe6.2, SdFe6.4, SdFe7.1, SdFe8.1, and SdFe11.1, have been identified in different populations belonging to the Andean and Mesoamerican gene-pool (Blair et al., 2010, 2011; Blair and Izquierdo, 2012; Cichy et al., 2014; Izquierdo et al., 2018; Katuuramu et al., 2018; Diaz et al., 2020). SdFe6.1 was only reported previously by Diaz et al. (2020) in a Mesoamerican Multi-parent Population and the positive allele came from a biofortified genotype MIB778. It was suggested that this allele in MIB778 may have been derived from a *Phaseolus dumosus* parent. There are no reports about SdFe3.1 but in this study, the QTL proved to be highly important for the analyzed biofortified populations.

For SdZn, 12 QTLs were identified, of which 6 have been reported previously. SdZn6.1 has been reported in several populations of both gene pools (Cichy et al., 2009; Blair et al., 2010; Blair and Izquierdo, 2012; Izquierdo et al., 2018; Katuuramu et al., 2018). SdZn3.1 and SdZn10.1 were only identified in an inter-gene pool bi-parental population (Astudillo et al., 2013) and an Andean Diversity Panel (Katuuramu et al., 2018). Conversely, SdZn2.3 and SdZn8.2 only were reported in a population belonging to the Mesoamerican gene pool (Freyre et al., 1998; Blair et al., 2009; Izquierdo et al., 2018; Diaz et al., 2020). Mapping certain QTL in different gene pools demonstrates the complex control over micronutrient accumulation in the common bean that can vary between the Andean and Mesoamerican genotypes. It has been reported that Andean genotypes showed higher Fe concentrations than Mesoamerican genotypes and that Mesoamerican lines presented higher Zn contents than Andean lines (Islam et al., 2002). The high degree of correspondence with other reports for both Fe and Zn lends credibility to the current results.

One of the main challenges that common bean breeders working on biofortification face is the negative correlation between iron accumulation and yield (Raatz, 2018). Genotypes with high iron accumulation have been associated with poor yield and lower yield components (fewer pods, fewer seeds, fewer seeds per pod, or poorly filled seed) (Beebe, 2020). This same tendency is reflected in the correlations presented in Figure 3 and Supplementary Table 3 and has been reported previously (Diaz et al., 2020). Out of 14 loci for high iron, 6 were associated with low values of yield components (SdFe6.2, SdFe6.4, SdFe7.1, SdFe9.1, SdFe11.1, and SdFe11.2). These loci could contribute as much as 39% of the total additive value for high iron. Similarly, out of 12 loci for high Zn, 6 were associated with negative effects on yield components (SdZn2.1, SdZn2.2, SdZn2.3, SdZn6.1, SdZn7.1, and SdZn8.2), summing up to 49% total additive effect for Zn concentration. Several of these “false” loci were reported by other authors, so this has been a problem in the genetic analysis of mineral concentration for several years. On the other hand, the QTLs SdFe3.2, SdFe5.1, SdFe6.1, SdFe6.3, SdFe7.2, SdFe8.1, and SdFe11.3 were not associated with loci related with yield components, making them attractive for breeding through marker-assisted selection (MAS) because their selection would not adversely affect the yield. Another advantage of these identified QTLs is that some of these loci have been found in populations belonging to both gene pools, making them useful for improving Andean or Mesoamerican genotypes. The sum of additive effects not associated with poor yield could contribute as much as 40 mg/kg Fe. Even though it is likely not possible to maintain the full additive effects of these loci through genetic recombination, ample variability exists without affecting yield. Similarly, there are QTLs for Zn that were not co-located with QTLs related to yield components (SdZn3.1, SdZn4.1, SdZn5.1, SdZn5.2, SdZn8.1, and SdZn10.1). These QTLs can be implemented in the breeding program, which theoretically could increase Zn seed concentration by 10 mg/kg. Nonetheless, if limits exist to raising Fe and/or Zn concentration with the QTL studied here, additional sources of genetic variability for high minerals are being explored. Beebe (2020) reported that interspecific progeny derived from Phaseolus species that evolved on alkaline soils might be more receptive to raising iron levels. Initial phenotypic results with these interspecific crosses are promising but have not been studied yet to determine their genetic makeup.

The observed negative associations of seed mineral concentration with PHI and Sd/Pd, and in two cases with 100SdW, probably reflect simple factors of concentration of minerals. This may be due to a dilution effect of Fe and Zn caused by high carbohydrate translocation to the grain, a typical characteristic of genotypes with high yield and PHI. This has also been reported in other crops, such as chickpea (Diapari et al., 2014). Conversely, if seed mass is reduced, then as the iron in the pods is translocated to the seed, the concentration in the seed will be increased arithmetically. Furthermore, where selection for high iron has received priority in breeding, this will have unconsciously limited yield potential. These results reveal many of the causes behind the persistent negative correlation of yield and high minerals that have been the greatest challenge in breeding biofortified beans.

A strong phenotypic correlation between iron and zinc concentrations was observed here and has been reported previously (Blair et al., 2009; Cichy et al., 2009). Izquierdo et al. (2018) reported loci that contributed to high concentrations of both elements. They identified a MetaQTL (MQTL) denominated “MQTL_Fe&Zn_6.2,” containing eight QTLs identified in previous studies, such as sources of favorable alleles for Fe and Zn in both gene pools. MQTL_Fe&Zn_6.2 displayed higher PVE (27%) compared with other MQTLs identified in the same study. However, in this work, only one locus common to SdFe and SdZn was found, and this was associated with low values of PdN. This suggests that the correlation of Fe and Zn concentration that has been observed here and elsewhere may be driven in part by poor pod formation and/or poor pod and grain filling. The hotspot 12 is the only locus in which QTLs controlling the accumulation of Fe and Zn were co-located on Pv06 (∼28–29 Mpb).

The processes of iron and zinc uptake, transport, metabolism, and storage in sinks of interest are regulated by complex genetic mechanisms, of which several genes have been identified as possible candidates regulating these processes (Connorton et al., 2017). MQTL_Fe&Zn_6.2 mentioned above is located with Hotspot 12 (SdFe6.4/SdZn6.1), which is near the gene model Phvul.006G196600 that codifies a basic helix-loop-helix DNA-binding protein (bHLH). This gene is homologous to bHLH105/ILR3, an *Arabidopsis* gene codifying a transcriptional activator of responses to Fe deficiency, that also downregulates the expression of ferritin genes, implicated in the control of Fe homeostasis (Samira et al., 2018; Tissot et al., 2019; Gao et al., 2020). FER-LIKE REGULATOR OF IRON UPTAKE (FER) is required for induction of iron mobilization genes in Arabidopsis thaliana (Bauer et al., 2007). Phvul.005G430500 is homologous to FER and is located near SdFe5.1 (∼36.8 Mpb). Phvul.011G211900 is located near SdFe11.3 and is homologous to FRD3, which has a function of root xylem loading of iron chelator and efficient uptake out of the xylem into sink cells in Arabidopsis (Green and Rogers, 2004). AtNRAMP6 is a gene codifying a multispecific vacuolar metal transporter and plays an important role in the growth of *Arabidopsis* in Fe-deficient conditions (Li et al., 2019). Phvul.005G182000 (corresponding to SdFe5.1) and Phvul.009G127900 (corresponding to SdFe9.1) are homologous to AtNRAMP6. Phvul.003G143800 is homologous of AHA2, a proton pump H+-ATPASE2 controlling acidification-reduction-transport of iron uptake in Arabidopsis (Campos, 2020). The Zrt and Irt-like protein (ZIP) is well characterized for its role in Zn and Fe transporter (Astudillo et al., 2013); Phvul.002G184200 (SdZn2.2), Phvul.008G259200 (SdZn8.2), Phvul.010G059200 (SdZn10.1), and Phvul.011G058500 (SdFe11.1) have been identified as possible candidate genes belonging to ZIP metal ion transporter family.

In terms of conventional breeding, successful cases have already been reported using these populations evaluated for obtaining varieties with high accumulation of Fe and acceptable yield in different conditions: BIO-101 in Colombia (83 mg/kg of Fe and 44 mg/kg of Zn),^3^ SMR100 in Nicaragua (89 mg/kg of Fe and 35 mg/kg of Zn),^4^ and INTA Bio-Apante in Nicaragua^5^ were biofortified lines that used RILs of SMC40 × SCR16 population as parental materials.

An effect of drought was observed only on yield components, while Fe and Zn accumulation was not affected by the drought stress. The positive correlation between yield components has previously been reported under drought conditions (Polania et al., 2016a,b). Regarding the invariable Fe and Zn contents under stress conditions, Smith et al. (2019) explained that seeds have an evolutionary tendency to ensure adequate resources for germination and present buffering effects to maintain similar nutrient concentrations in drought and irrigated conditions, but the explicit mechanisms are not clear.

Pod harvest index expresses the ratio between the biomass in seed and pod and is a useful indicator of the remobilization of photosynthates to seed (Assefa et al., 2013). However, until now, it is not clear yet which of these two components have the key effect on PHI, and if there is a possible genetic relationship that explains the different behavior of PHI in each genotype. In this study, a stronger phenotypic correlation was observed between PHI with Sd/Pd and SdN than with PdN in all populations. However, the phenotypic correlation of PHI with 100SdW was low or not significant in this study (Figure 3), similar to results reported by Diaz et al. (2020) and Berny Mier Y Teran et al. (2019). Berny Mier Y Teran et al. (2019) suggested that sink strength due to the number of seeds is higher than sink strength based on the seed size. This could indicate that a key factor that affects PHI is the number of seeds rather than seed weight itself. In the genetic analysis, most QTLs identified for the number of seeds per pod (Sd/Pd) were co-localized with QTLs identified for SdN, and Sd/Pd has a high positive correlation with PHI. Moreover, all QTLs of PdN having a positive additive effect were associated with the SMC allele, which was parental with high micronutrient concentration but poor pod filling.

The main QTL found in this study for PHI (PHI 7.2), with the highest PVE and additive effect for the trait (Table 2), has been reported previously in Mesoamerican populations (Diaz et al., 2018; Berny Mier Y Teran et al., 2019) and in an Andean population (Sedlar et al., 2020), and could be used for breeding in both gene-pools. PHI1.1 and PHI8.1 have been identified previously in a Mesoamerican MAGIC population (Diaz et al., 2020). PHI1.2, PHI5.1, and PHI7.3 have been described in previous studies in Mesoamerican bi-parental populations (Asfaw et al., 2012; Diaz et al., 2018; Berny Mier Y Teran et al., 2019). PHI5.2 was reported by Sedlar et al. (2020) in an Andean bi-parental population. These QTLs seem to be independent of micronutrients content loci and could be used for MAS and theoretically could enhance PHI values by >20%, which discovers these markers very promising for breeding for drought tolerance and/or high yield. Other QTLs for other yield related-traits have also been reported, Berny Mier Y Teran et al. (2019) reported a QTL for seeds per pod near to Sd/Pd1.1. Similarly, Sedlar et al. (2020) reported a QTL near to Sd/Pd2.1, the QTL with the highest PVE. Other QTLs, such as PdN2.2, PdN4.1, SdN2.1, and SdN6.1, were reported previously in other Mesoamerican bi-parental populations (Diaz et al., 2017). Mukeshimana et al. (2014) found a QTL near to PdN8.2, this QTL was present in multiple trials.

One issue of PHI that has been discussed little is its role as a domestication trait associated with greater partitioning to grain, and whether the selection of genotypes with better pod filling was linked to other traits related to the domestication of common beans, such as pod indehiscence and determinate growth habit. PHI5.2 and PHI8.1 were co-located in the same genomic regions that controlled pod fiber or dehiscence for common bean (Parker et al., 2020). In the same way, PHI1.2 and PHI1.3 are near to the *fin* locus, a key region that controlled determinacy and photoperiod sensitivity in common bean (Kwak et al., 2012). This could indicate that PHI reflects traits that were enhanced in domestication and that loci associated with PHI could be related to domestication.

Understanding the segregation variation between the populations. Heterozygosity information in the populations is presented (Supplementary Figure 5). The utility of the early generation’s higher variability could be implemented as sources as a pedigree follow-up. In consequence, the analysis of the use of F_3_ and F_4_ populations was mandatory previously for further selection cycles.

## Conclusion

This study evaluated the phenotypic and genetic correlation between Fe/Zn accumulation and yield components using QTL analysis in three biofortified populations developed in the CIAT bean-breeding program. The results in this work validate the negative phenotypic correlation previously described between yield and Fe/Zn accumulation in phenotypic terms. In addition, we are identifying several loci controlling both traits. We hypothesize this strong correlation is a dilution effect of Fe and Zn caused by high carbohydrate translocation to grain. Conversely, loci associated with high minerals and low values of yield components should be avoided. On the other hand, we observed QTL for Fe/Zn segregating independently of the other QTL for yield components, possibly mediated by transporters with affinity to minerals. The selection of these regions will enable enhanced levels of Fe/Zn and should not affect yield performance. QTLs observed in this and previous studies suggest similar genetic control in both gene pools at some loci and these QTLs show a high potential to be implemented in a breeding program focused on biofortification.

## Data Availability Statement

The original contributions presented in the study are publicly available. This data can be found here: https://dataverse.harvard.edu/dataset.xhtml?persistentId=doi:10.7910/DVN/LI31YG.

## Author Contributions

SB conceived the study and designed the populations. CC and MG carried out the breeding populations advancement. JP did the field trial evaluation. SD did the phenotypic data analysis and linkage analysis. BR supervised the molecular laboratory. SD and DA-S performed the genotypic data analysis. SD and SB wrote the manuscript. All authors read and approved the final version of the manuscript.

## Conflict of Interest

The authors declare that the research was conducted in the absence of any commercial or financial relationships that could be construed as a potential conflict of interest.

## Publisher’s Note

All claims expressed in this article are solely those of the authors and do not necessarily represent those of their affiliated organizations, or those of the publisher, the editors and the reviewers. Any product that may be evaluated in this article, or claim that may be made by its manufacturer, is not guaranteed or endorsed by the publisher.

## Footnotes

1
http://www.cropontologycurationtool.org/

2
https://hudsonalpha.org/

3
https://lac.harvestplus.org/en/new-biofortified-bean-varieties-bio-101-bio-107/

4
https://inta.gob.ni/wp-content/uploads/2019/11/Brochure-Frijol-Biofortificado-HarvesPlus-2019.pdf

5
https://lac.harvestplus.org/ometepe-biofortificados-nicaragua/

## Supplementary Material

Supplementary Figure 1 Precipitation, maximum, and minimum temperatures during trials at Palmira, Colombia.

Supplementary Figure 2 Histograms of frequency distribution for Fe and Zn in seven trials of the SCR16 × SMC40, SMC33 × SCR16, and SMC44 × SCR9 populations.

Supplementary Figure 3 Genome-wide association analysis for Fe accumulation (SdFe) showing Manhattan and Q-Q plot for SMC × SCR populations with 24,785 markers in seven trials. The Bonferroni correction threshold (*p* = 0.05) is presented as a red horizontal line based on the number of markers for the population.

Supplementary Figure 4 Genome-wide association analysis for Zn accumulation (SdZn) showing Manhattan and Q-Q plot for SMC × SCR populations with 24,785 markers in seven trials. The Bonferroni correction threshold (*p* = 0.05) is presented as a red horizontal line based on the number of markers for the population.

Supplementary Figure 5 Heterozygosity of individuals and heterozygosity of markers in the SMC × SCR populations.

Supplementary Table 1 Phenotypic variability and broad-sense heritability (H^2^) for evaluated traits in the trials 2011, 2012, 2013, 2014, and 2015 of the SCR16 × SMC40, SMC33 × SCR16, and SMC44 × SCR9 populations.

Supplementary Table 2 Component variance, F statistic evaluated for seven trials to SdFe, seed iron; SdZn, seed zinc; PHI, pod harvest index; PdN, pod number; SdN, seed number; Sd/Pd, number of seeds per pod; 100SdW, 100 seed weight in SCR16 × SMC40, SMC33 × SCR16, and SMC44 × SCR9 populations.

Supplementary Table 3 Pearson’s correlations coefficients between evaluated traits on three populations SCR16 × SMC40, SMC33 × SCR16, and SMC44 × SCR9 for each trial and each trait. Significance of correlations indicated as ^***^*p* < 0.0001; ^**^*p* < 0.001; **p* < 0.01; ns, not significant. SdFe, seed iron; SdZn, seed zinc; PHI, pod harvest index; PdN, pod number; SdN, seed number; Sd/Pd, number of seeds per pod; 100SdW, 100 seed weight.

Supplementary Table 4 Details of all QTL identified by inclusive composite interval mapping for additive effects (ICIM-ADD), genetic and physical position, LOD, phenotypic variation explained mapped for six traits in SCR16 × SMC40, SMC33 × SCR16, and SMC44 × SCR9 populations evaluated in 2011, 2012, 2013, 2014, and 2015.
