# Supplementary material for: Genetic Correlation Between Fe and Zn Biofortification and Yield Components in a Common Bean (Phaseolus vulgaris L.)
Source: Front Plant Sci. 2022 Jan 3;12:739033. doi: 10.3389/fpls.2021.739033 (PMC8761845; doi:10.3389/fpls.2021.739033)
Supplement: Supplementary Figure 1 — Precipitation, maximum, and minimum temperatures during trials at Palmira, Colombia. [file Data_Sheet_1.zip › Data Sheet 1/Supplementary Figure 1.PDF]

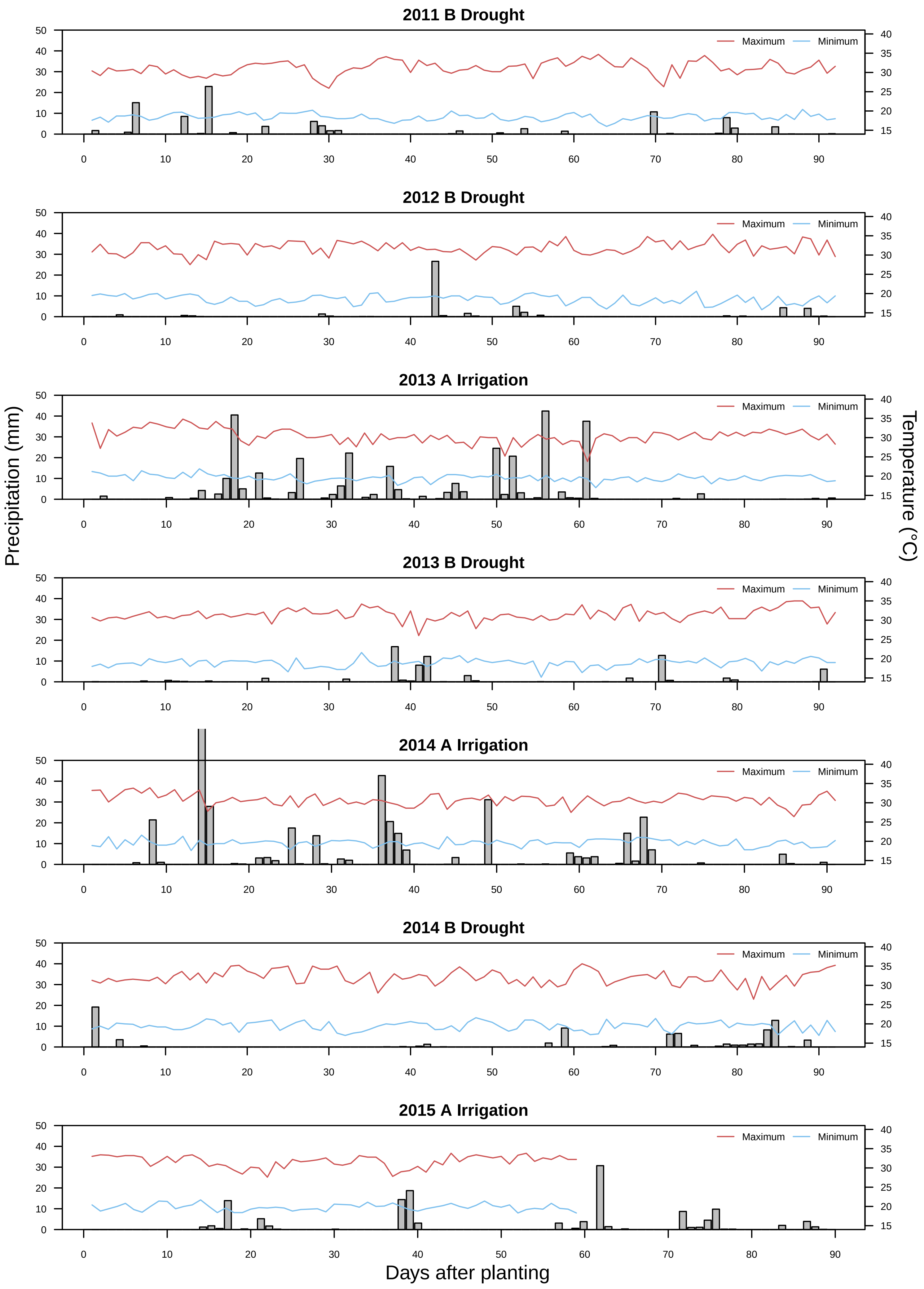
2011 B Drought
2012 B Drought
2013 A Irrigation
2013 B Drought
2014 A Irrigation
2014 B Drought
2015 A Irrigation
Maximum
Minimum
Precipitation (mm)
Temperature (°C)
Days after planting
