# Supplementary figures and images for: Genetic Correlation Between Fe and Zn Biofortification and Yield Components in a Common Bean (Phaseolus vulgaris L.)
Source: Front Plant Sci. 2022 Jan 3;12:739033. doi: 10.3389/fpls.2021.739033 (PMC8761845; doi:10.3389/fpls.2021.739033)

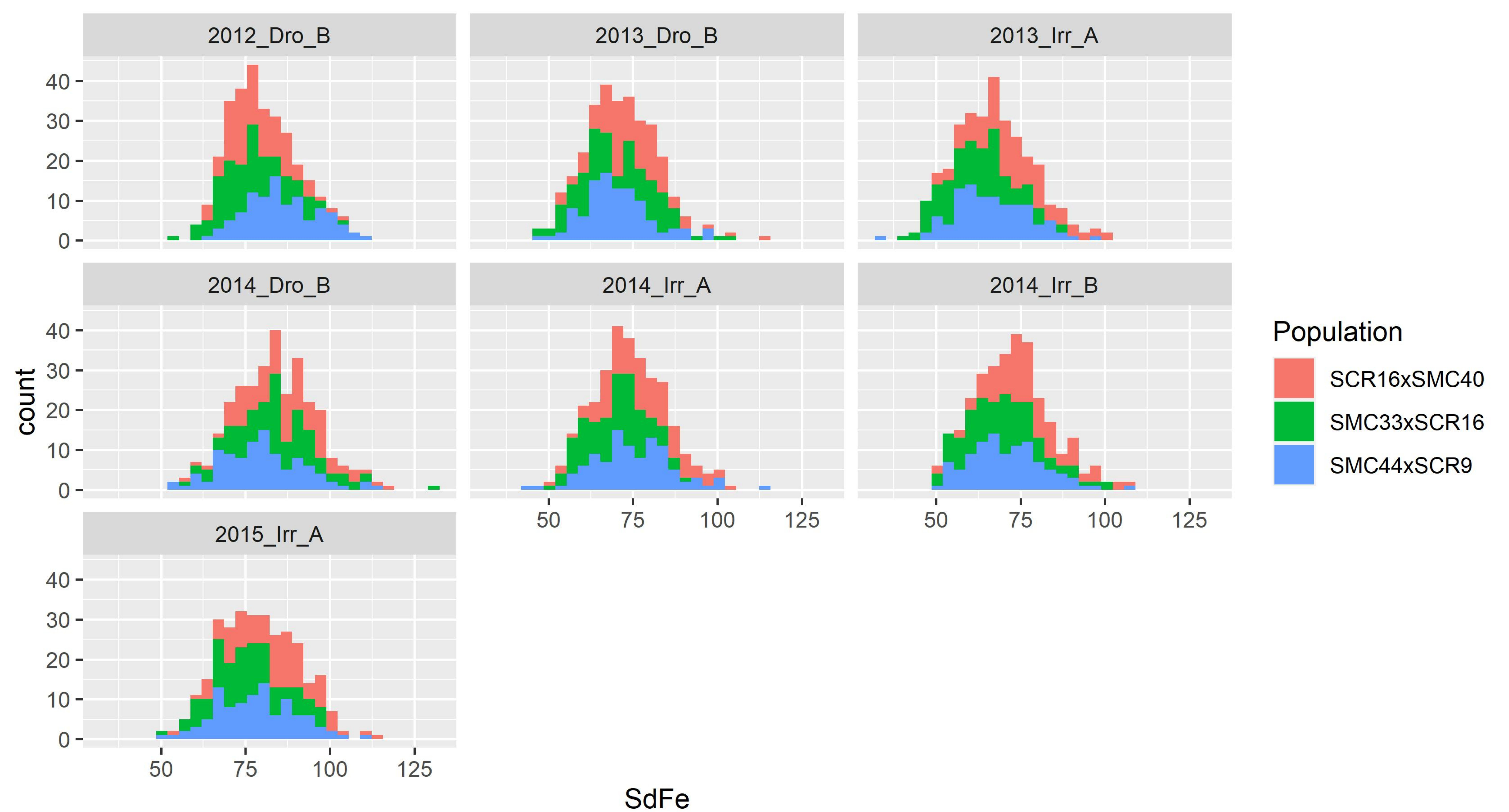

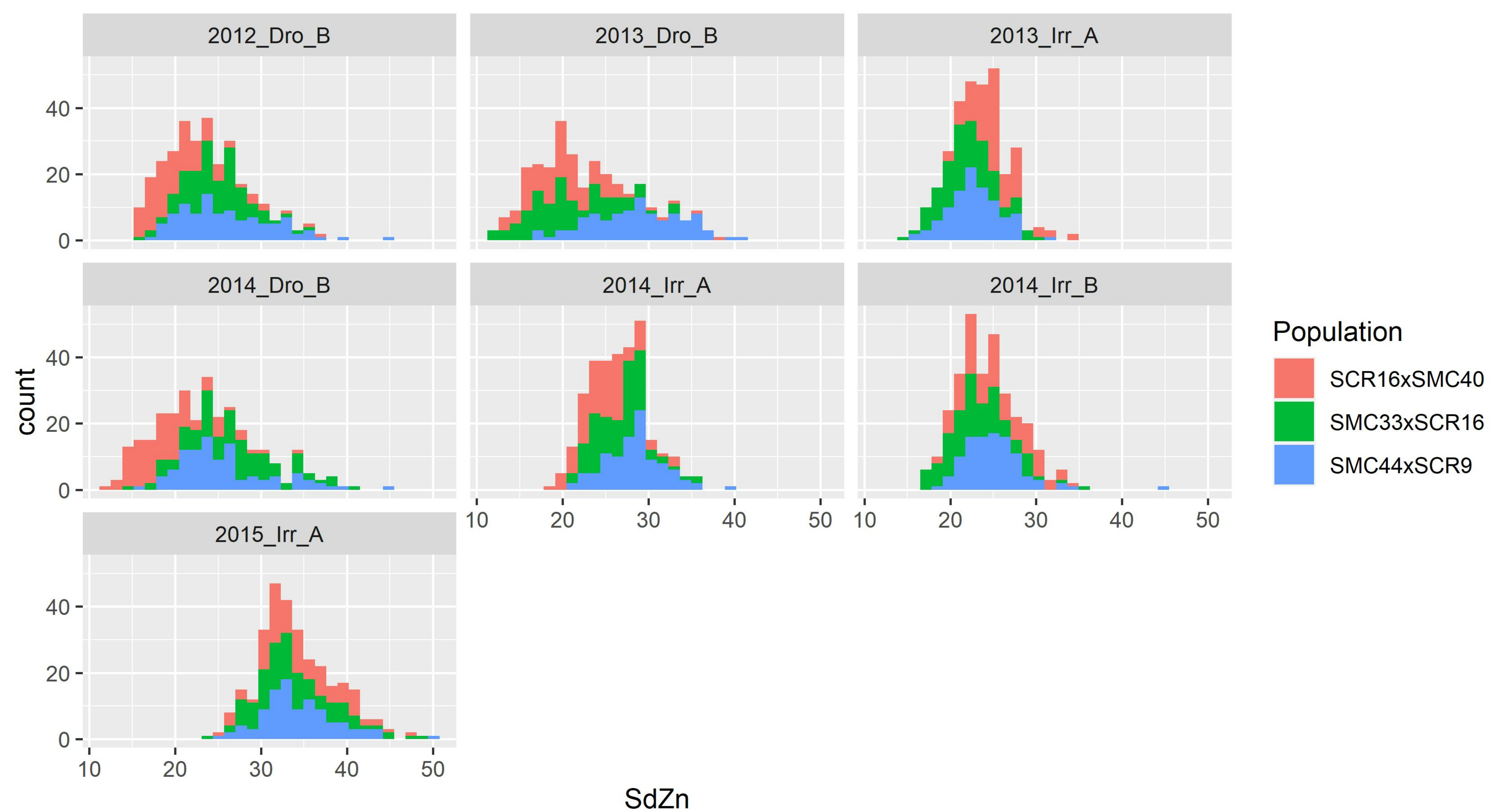

Supplement: Supplementary Figure 1 — Precipitation, maximum, and minimum temperatures during trials at Palmira, Colombia. [file Data_Sheet_1.zip › Data Sheet 1/Supplementary Figure 2.pdf]

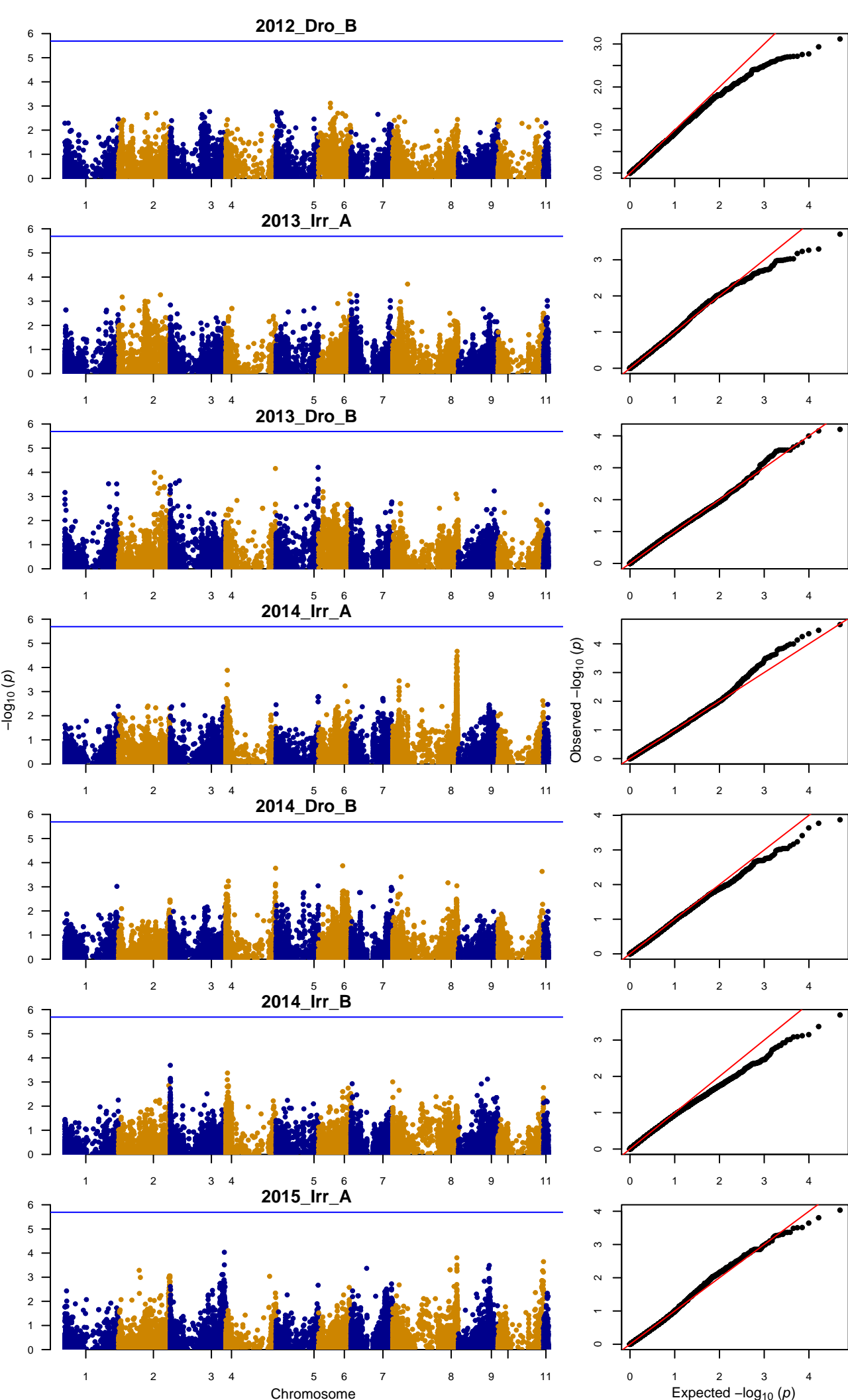

Supplement: Supplementary Figure 1 — Precipitation, maximum, and minimum temperatures during trials at Palmira, Colombia. [file Data_Sheet_1.zip › Data Sheet 1/Supplementary Figure 3.PDF]

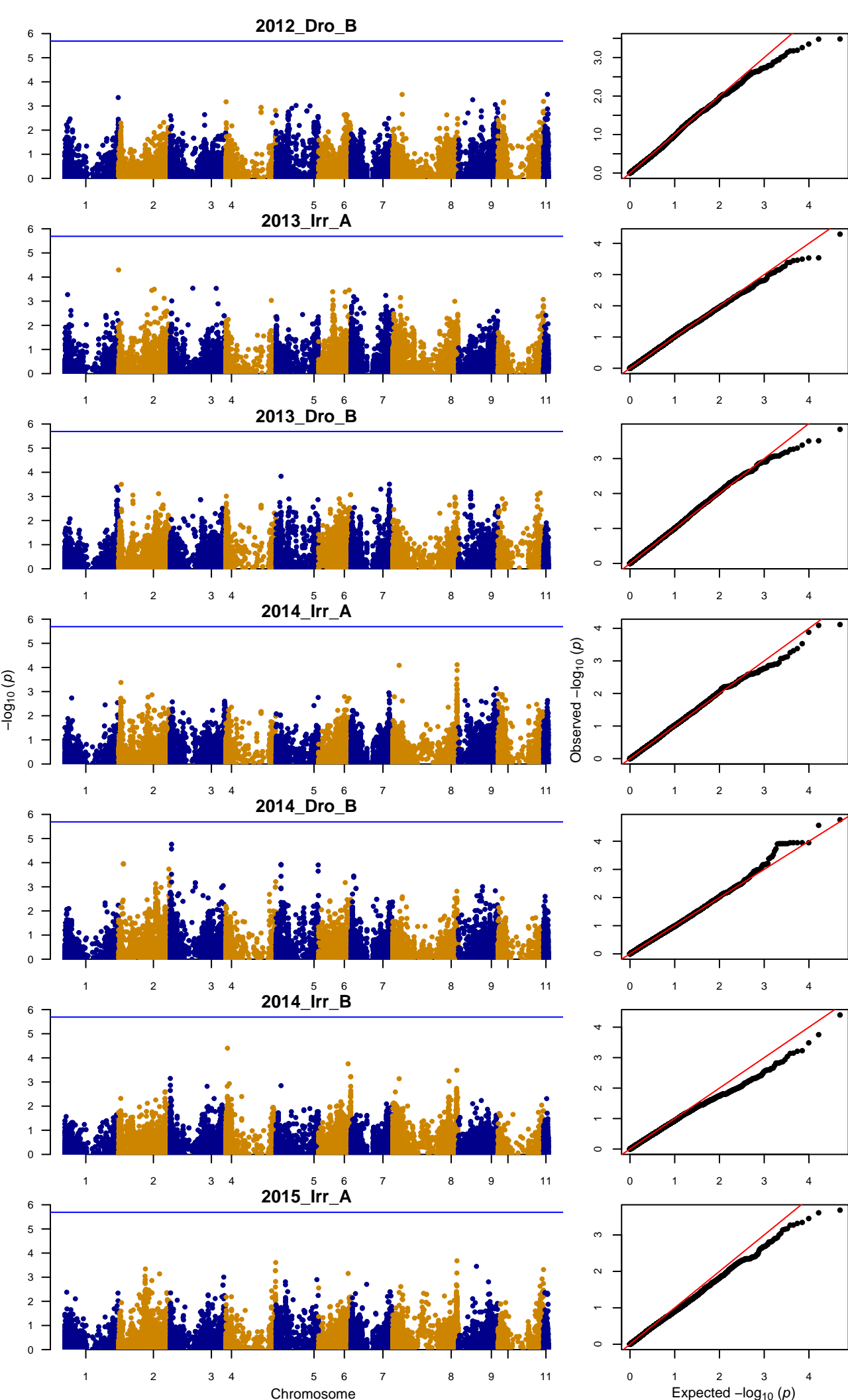

Supplement: Supplementary Figure 1 — Precipitation, maximum, and minimum temperatures during trials at Palmira, Colombia. [file Data_Sheet_1.zip › Data Sheet 1/Supplementary Figure 4.PDF]

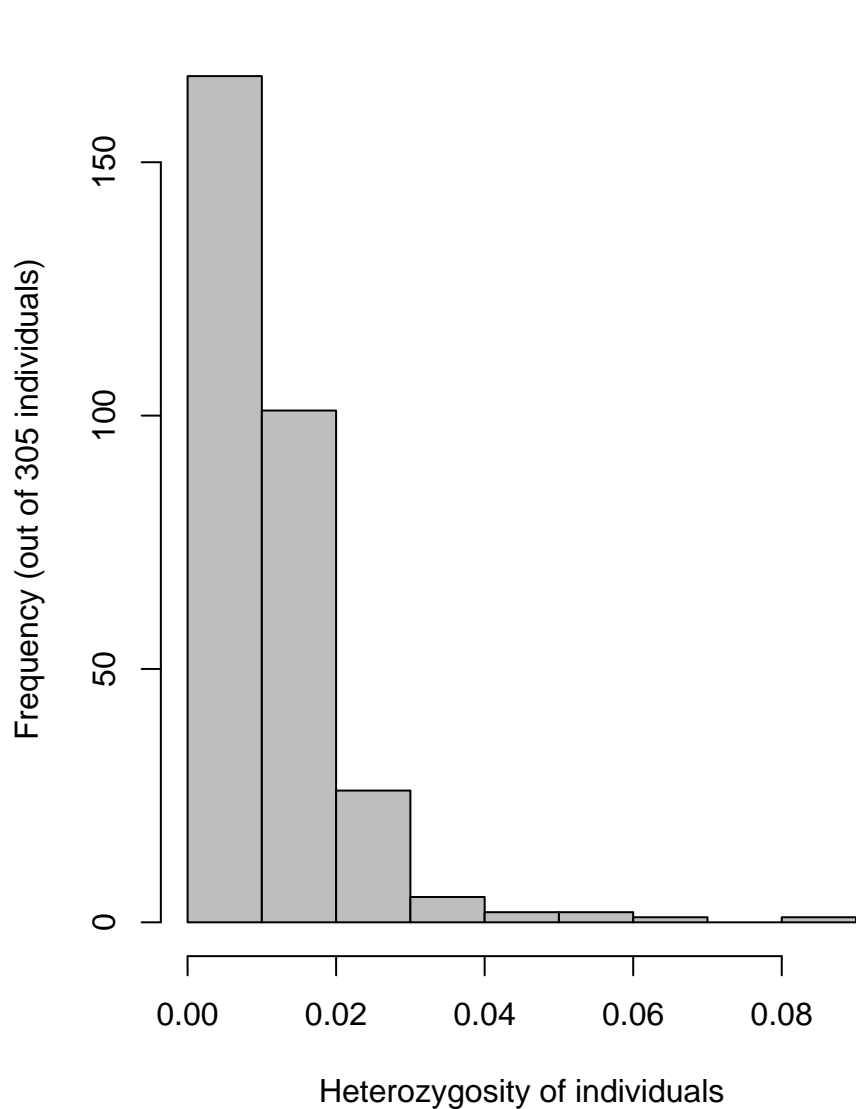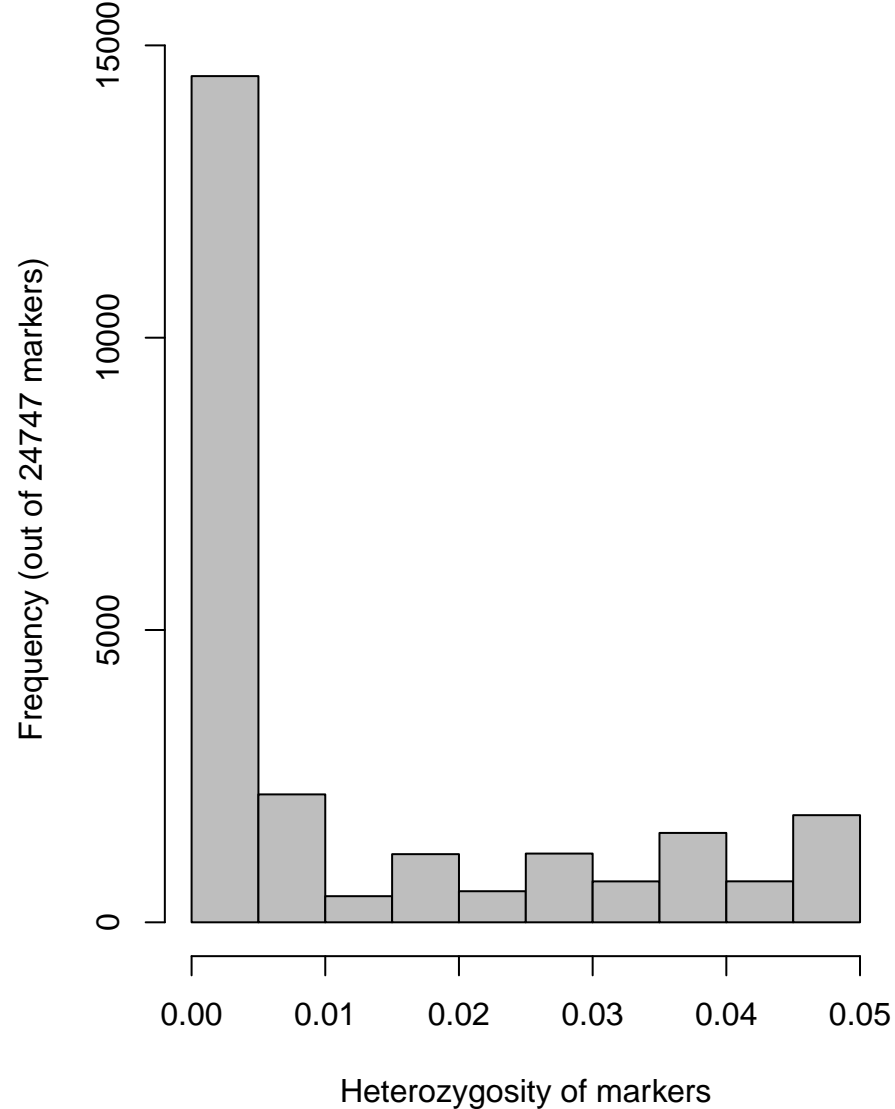

Supplement: Supplementary Figure 1 — Precipitation, maximum, and minimum temperatures during trials at Palmira, Colombia. [file Data_Sheet_1.zip › Data Sheet 1/Supplementary Figure 5.PDF]
